# Urban endoliths: incidental microbial communities occurring inside concrete

**DOI:** 10.3934/microbiol.2023016

**Published:** 2023-03-30

**Authors:** Jordan Brown, Corona Chen, Melania Fernández, Deborah Carr

**Affiliations:** 1 Department of Biological Sciences, Texas Tech University, Lubbock, Texas, USA; 2 The University of Chicago Laboratory School, Chicago, Illinois, USA; 3 Department of Plant and Soil Science, Texas Tech University, Lubbock, Texas, USA; 4 Lankester Botanical Garden, University of Costa Rica, Cartago, Costa Rica

**Keywords:** building materials, built environments, global change, urban ecology, cryptic ecosystems, lithobiont, biosignatures

## Abstract

Concrete is now a prevalent type of synthetic rock, and its production and usage have major environmental implications. Yet, assessments of ordinary concrete have rarely considered that concrete itself is potential habitat for a globally important microbial guild, the endolithic microbes, which live inside rocks and other mineralized substrates. We sought evidence that many common concrete structures harbor endolithic microbial communities and that these communities vary widely depending on the conditions imposed by the concrete. In Summer 2022, we obtained samples from various concrete structures found throughout Lubbock, Texas, USA and subjected the internal (non-surface) portions of each sample to controlled microbial life detection tests including culture tests, DNA quantifications, DNA amplification tests, and ATP assays. The great preponderance of positive life detection results from our concrete samples suggests that most modern concrete hosts cryptic endolith communities composed of bacteria, sometimes co-occurring with fungi and/or archaea. Moreover, many of these microbes are viable, culturable, and identifiable via genetic analysis. Endolith signatures varied widely across concrete samples; some samples only yielded trace evidence of possibly dormant microbes while other samples contained much more microbial biomass and diversity, on par with some low-biomass soils. Pre-cast masonry units and fragments of poured concrete found underwater generally had the most endolith signatures, suggesting that concrete forms and environmental positioning affect endolithy. Endolith biosignatures were generally greater in less dense and less alkaline concrete samples. So, concrete endolith communities may be as ubiquitous and diverse as the concrete structures they inhabit. We propose further research of concrete endoliths to help clarify the role of modern concrete in our rapidly urbanizing biosphere.

## 1. Introduction

### 1.1. Concrete: the rock made by humans

In many tangible ways, the modern world has been built with concrete. Being a cheap, workable, and open-ended composite material, poured and pre-cast concrete is used for everything from setting fence posts to building massive infrastructures such as highways and dams. It has become the most abundant human-made material on Earth, with production rates far exceeding that of any other building material including steel and wood [1] and cumulative global quantities exceeding 549 gigatons [2]. The vast, almost unimaginable scale of concrete usage has made it a ‘hyperobject’ [3] unlikely to be replaced in the foreseeable future [4]. It is therefore important to study all aspects of this ubiquitous material.

Concrete has a long and intensive history of scientific study and entrepreneurial innovation, mostly aimed at improving its material properties and structural utility [4]. More recently, researchers have been assessing the cement and concrete industries, specifically in terms of the industries' resource consumption [5], greenhouse gas emissions [6], health impacts [7], economic importance and market trends [8], costs and benefits during natural disasters [9] and potential for recycling and circularity [10]. In recent decades, concrete material science and environmental research have converged into the prevalent but still largely unexplored field of ‘green’ or ‘sustainable concrete’ [11]. In the interest of sustainable concrete, specialized concrete mixes have been designed to contain certain mineral precipitating bacteria and fungi, which endow concrete structures with self-repair properties throughout their volumes, thus reducing the need for active maintenance and delaying replacement [12],[13]. However, these biology-based innovations have not incorporated the idea that various microbes can incidentally inhabit the internal volumes of ordinary concrete.

### 1.2. Concrete as microbial habitat

Imagining the insides of concrete as microbial habitat first requires understanding that concrete is synthetic rock, analogous to naturally occurring conglomerate rocks [14]. Many natural rocks and mineral formations are now known to harbor cryptic microbes called ‘endoliths.’ Endoliths comprise a diverse set of organisms (mostly bacteria, fungi, archaea, and algae) that are able to live inside solid rock in various ways, filling lesser-known ecological niches that nonetheless cover an enormous fraction of the biosphere, including the so-called ‘deep biosphere’ [15]. Earth is a rocky planet with relatively thin layers of water, soil, and atmosphere, and through specialized life-history strategies or incidental adaptations that allow survival inside rock, endoliths can take advantage of a large portion of the planet, including surface rocks and subsurface geologic layers nearly 5 km deep [16]. Endolithic ecosystems usually have relatively little biomass and low rates of biological activity, but the sheer size of the habitat they are able to exploit means that endoliths collectively influence Earth system processes, such as rock weathering [17], soil formation [18], and carbon cycling [19]. Endoliths in natural rocks give great precedence to the idea of cryptic ‘concrete endoliths,’ which can exploit Earth's ever-increasing supply of concrete in ways that are largely unexpected and unnoticeable. Luckily, a few have studied the endolithic microbes existing within the anthropogenic rock type we call concrete.

Coombes et al. [20] observed experimental concrete blocks being shallowly colonized by marine euendoliths (endoliths that can actively bore into rock and minerals). Maresca et al. [21], despite the chemistry-related challenges, successfully extracted and sequenced bacterial DNA from the insides of a set of concrete test cylinders, thereby developing a possible method for diagnosing internal microbial biodegradation in concrete. Following that study, Kiledal et al. [22] examined seasonal changes in bacterial communities inside concrete test cylinders and noted differences between two related types of concrete, thus elucidating some ecological aspects of concrete endoliths. These studies provide foundational support for a ‘concrete endolith hypothesis,’ but these did not test hypotheses about the ubiquity and variability of both prokaryotic and eukaryotic endoliths within diverse built environments. In the context of urbanization and the various trends of global change associated with concrete, it is now important to devote more study to the cryptic biology of concrete so that we can more completely understand concrete's environmental impacts. The first steps towards this goal include understanding how frequently endolithic bacteria, archaea, and fungi inhabit the various types of concrete found in our built environments.

### 1.3. Objectives and hypotheses

Our primary objective here was to survey common forms concrete gathered from various settings within a city environment for endolithic life, then use the survey results to test some basic hypotheses about concrete endoliths. Concrete is as variable as it is ubiquitous, so instead of conducting narrow analyses of particular types of concrete in select settings, we performed a broad microbiological investigation on a wide variety of real-world concrete structures. We designed this survey to determine if and how frequently the concrete present in our built environments is naturally inhabited by endolithic bacteria, fungi, and archaea. Although there is compelling evidence that the internal portions of concrete structures or ‘endo-concrete’ environments can host at least some types of microbes in certain capacities, there is still relatively little evidence of concrete endoliths, and there are several reasons to think that most concrete is inhospitable to microbes. So, we further tested the ‘concrete endolith hypothesis’ by noting if and when we obtained positive life-detection results from our concrete samples. We reasoned that, if concrete is suitable habitat for endolithic microbes, then we should be able to cultivate microbes and/or successfully detect biosignature molecules from at least some endo-concrete samples.

Assuming that we would obtain positive life-detection results during our survey, we also tested the ‘endolith-concrete interaction hypothesis.’ This stated that endolithic life detection results inside concrete will vary by the physicochemical characteristics of the respective concrete substrates and/or by the general environments and forms in which the concrete existed because concrete conditions affect resident microbes. So, we tested for significant differences in life detection results (culture test frequencies and biosignature molecule quantities) among different categories of concrete and for significant relationships between biological variables and concrete physicochemical variables.

To supplement our survey, we explored using scanning electron microscopy (SEM) for life detection. While unable to incorporate SEM micrographs into our main survey, we deemed it worthwhile to search for visual evidence of endoliths in a subset of our concrete samples. Such evidence would help corroborate the main life detection tests and help determine the feasibility of SEM in visualizing rock-embedded microbes without significant sample manipulation.

Lastly, we hoped to verify fungi among the viable microbes that exist deep within ordinary concrete. To our knowledge, there is no evidence of incidental endolithic fungi in ordinary concrete, but like many other microbiomes, endo-concrete microbiomes may include both prokaryotic and eukaryotic constituents, with important implications for how these communities may function. Our survey positioned us to cultivate fungi along with other endo-concrete microbes, so we will attempt to verify that some of the microbes we culture are indeed fungi using DNA sequencing.

## 2. Materials and methods

### 2.1. Concrete sample collection

We collected concrete samples throughout Lubbock, Texas, USA (33.5° N, 101.8° W, ~978 m elevation) between June–August 2022. Lubbock is in the Southern Great Plains region and has a semi-arid climate with a mean annual precipitation of 485 mm, a mean annual low temperature of 8.3 °C, a mean annual high temperature of 23.3 °C, and a mean annual temperature of 15 °C [23]. We collected 25 independent samples of concrete that we categorized as submerged poured concrete permanently underwater, belowground poured structures surrounded by soil, ground-level poured slabs with exposed top surfaces and bottom surfaces in contact with the soil, aboveground poured structures with no direct contact to the ground, or aboveground concrete masonry units (CMUs) which are pre-cast forms, some of which are colloquially known as cinder blocks (Table 1). These categories do not encompass all types of modern concrete nor do they represent the full range of historical and environmental factors that could affect concrete's ability to harbor microbes. Instead, we based this categorization scheme on what we could confidently know about each sample (i.e., the general way it had been formed and where it had primarily been prior to collection) while allowing for considerable variation within categories in terms of other factors (age of concrete, placement indoor vs. outdoor, with or without reinforcement, admixtures, or painted surfaces, etc.). Still, our final samples met several criteria.

We did not collect samples without permission or samples whose basic origins and histories we could not ascertain through personal communication with vested parties. We restricted our samples to ‘ordinary’ or ‘common’ concrete made with Portland cement (the most common concrete binder). We did not collect concrete made with highly specialized concrete mixes or samples of pure mortar, nor did we collect samples of asphalt, brick, or other similar materials. The bulk samples were typically between 10–25 kg and had an effective thickness of > 60 mm, ensuring that we could extract an adequate number of sub-samples of sufficient size. It was not feasible to collect samples that were actively in use, so we collected leftover and recently discarded concrete (often from demolition sites).

**Table 1.** Descriptions of 25 concrete samples collected throughout Lubbock, Texas, USA during June–August 2022.

Concrete sample category	Sample ID	Sample descriptions
	Submerged-1	Fragment (with re-bar) fallen from an old footbridge into a perennial freshwater stream
	Submerged-2	Fragment (with re-bar) fallen from an old culvert crossing into a perennial freshwater stream
Submerged fragment (poured)	Submerged-3	Discarded fragment left underwater near the shore of a freshwater pond
	Submerged-4	Discarded pavement fragment left underwater in an urban wetland area
	Submerged-5	Recycled fragment (with re-bar) inside a caged retaining wall at the edge of a freshwater pond

	Belowground-1	Building footing (with re-bar) excavated during a large-scale renovation
	Belowground-2	Concrete junction box excavated during a building's plumbing upgrade
Belowground structure (poured)	Belowground-3	Post setting recently unearthed from a parking lot
	Belowground-4	Post setting recently unearthed from an urban sidewalk
	Belowground-5	Post setting recently unearthed from grassy area in an urban park

	Ground-1	Sidewalk in a residential area, directly in front of a single unit house
	Ground-2	Housing pad (with re-bar) beneath a recently demolished large house
Ground-level slab (poured)	Ground-3	Sidewalk in the courtyard of an apartment complex
	Ground-4	Sidewalk (with re-bar) between a large commercial building and a large parking lot
	Ground-5	Sidewalk between a small commercial building and a street

	Aboveground-1	Indoor flooring (with re-bar) from a high-rise office building
	Aboveground-2	Floor of outdoor patio (with re-bar) extending from a 4th-story hotel room
Aboveground structure (poured)	Aboveground-3	Partially painted floor (with re-bar) of an open-style, multi-story parking garage
	Aboveground-4	Top layer of a short dividing wall between two parking lots
	Aboveground-5	Dislodged fragment from the top of an elevated street curb

	CMU-1	8-inch, 2-core, regular-style cinder block left outside in a pile atop a concrete slab
	CMU-2	8-inch, 2-core, regular-style cinder block used to hold planters in a greenhouse
Aboveground concrete masonry unit (pre-cast)	CMU-3	8-inch, 3-core, regular-style cinder block left in a pile in a forested urban park
	CMU-4	4-inch, 3-core, painted cinder block from a demolished block and mortar wall
	CMU-5	8-inch, 2-core, regular-style concrete block leftover from a recently built outdoor wall

### 2.2. Extracting, cutting, pulverizing, and sieving of concrete samples

From each large bulk sample of poured concrete, we extracted several sub-samples as cylindrical cores (38 mm diameter, at least 65 mm long) using a drill press and a diamond coring bit. We avoided coring from the edges of the bulk samples and trimmed 5 mm off the top of each core and at least 5 mm from its bottom with a tile saw, leaving a 50 mm core that contained none of the original surface portions. From each pre-cast CMU sample, we cut out several cuboids from the face shells with a tile saw and trimmed off 5 mm from the tops and bottoms. When we trimmed off 5 mm from the surfaces of our samples, we removed material that was likely inhabited by endoliths, particularly endoliths that colonized the concrete after it solidified, However, for this study, we wanted to analyze the endolithic zone of the concrete samples without the confounding effects of the epilithic surface portions. Additionally, we extracted one cylindrical core from the corner of each CMU for compressive strength analysis (discussed below). As an initial sterilization step, we wiped all sub-samples designated for biological analysis with 95% ethanol immediately after trimming and then stored these temporarily in clean plastic bags.

**Figure 1.**
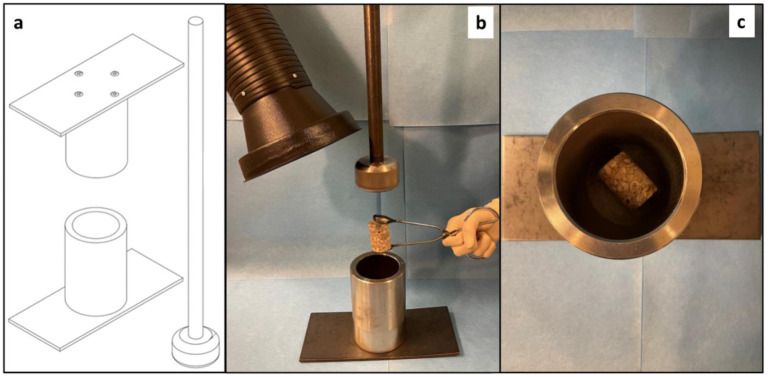
The setup for aseptically pulverizing concrete samples. Note: (a) Schematic diagram of the pulverizer: a custom-made, stainless steel, fully autoclavable, mortar and pestle-style instrument consisting of a base plate affixed with a 13-cm deep, 8-cm wide cylindrical sample receptacle (top- and bottom-view shown) into which a plunger is vertically inserted. (b) A prepared concrete sample being placed into the pulverizer base while it is positioned under a portable fume extractor and as the plunger is ready to begin pulverizing. (c) A typical concrete sample resting in the receptacle of the pulverizer; this receptacle holds two large, 200-gram concrete samples.

One sub-sample (core or cuboid) from each bulk sample was pulverized to make it suitable for biological and chemical analysis. We pulverized each sub-sample with a stainless-steel mortar and pestle custom-made to crush large, rocky samples to various particle sizes while minimizing contamination (Figure 1). We sterilized the pulverizer before each use by scrubbing it with soap and water, rinsing it thoroughly with purified water, wiping it with 95% ethanol, autoclaving it at 121 °C for 30 minutes, then letting it cool to room temperature. We positioned the pulverizer on the floor directly under the snorkel of a portable fume extractor set to maximum suction, which maintained a strong upward draft away from the base of the pulverizer and prevented any airborne contaminants from settling onto the pulverizer. Immediately prior to inserting a concrete sub-sample into the pulverizer, we performed a final surface sterilization on the sub-sample to remove any contaminants acquired during extraction, trimming, and storage. We did this by twice repeated flame sterilization (briefly coating the sub-sample in 100% ethanol and then igniting the ethanol until it burned away completely). Immediately after surface sterilizing, we placed the sub-sample in the pulverizer, manually crushed the sample for ~5 minutes to produce a mixture of fine and coarse particles, then transferred the entire pulverized sample to a sterile storage container. We developed this pulverization method when other devices (standard mortar and pestles, ring and puck mills, and clean benches) proved unsuitable for crushing samples of the sizes we required (for this study and others) and in a manner that reliably produced fine and coarse particles. We considered that pulverizing samples in this way might reduce the viability of some microbes and the stability of some biomolecules, but we assumed that this method would not critically damage microbes and biomolecules because high-quality DNA has been isolated from concrete after much more vigorous sample grinding [21].

After the sub-samples were pulverized, we sieved each sample in a laminar flow hood. We passed the sample through a sterilized, stainless steel, 1-mm sieve and reserved both the fine (< 1 mm) and coarse (1–10 mm) fractions in separate sterile containers. We stored the fine fractions at −20 °C and the coarse fractions at 4 °C until subsequent analyses. We left other sub-samples and portions of each bulk sample intact for different analyses.

### 2.3. Physicochemical analyses of concrete samples

We used a large tile saw to expose a complete cross-section of each bulk sample. We sprayed a 1% phenolphthalein pH indicator solution onto the freshly exposed face and noted the color change inward from the surfaces of the sample as a measure of carbonation depth. Although sometimes prone to underestimates, the carbonation depths shown after phenolphthalein application reveal the spatial extent to which acidifying carbonation reactions have gradually penetrated the otherwise highly alkaline concrete structure from the outside in, bringing some of the outermost material to a pH of 8.6 or below [24],[25]. We calculated a mean carbonation depth for each bulk sample by measuring the distances to the carbonation front (mm) from 3–5 haphazardly selected points along the cross-section. We measured inward from the same surface from which we extracted sub-samples and to the depth that any hue of purple was visible (though we sometimes observed various hues of purple within the same cross-section). Because our samples varied greatly in terms of size and shape and included both poured forms and pre-cast CMUs, we calculated a relative and standardized measure of carbonation from the absolute carbonation depths which we call carbonation proportion (the portion of the associated sub-samples that was carbonated, expressed as a decimal proportion between 0 and 1). For poured samples, the sub-samples were cores whose bottoms reached 55 mm into the inside of the bulk sample. For CMU samples, the primary sub-samples were extracted from the entire face shells, which were generally 30–40 mm thick. We reported samples with no highly alkaline regions (no color change to purple in the profile) or with only small, scattered patches of purple alkaline material within their sub-sample zone as fully carbonated (carbonation proportion of 1.0) and samples with no acidified regions within their sub-sample zone (all purple) as having carbonation proportions of 0.

Prior to any pulverization, we measured the mass, length, width, height, and/or diameter (mm) of each cylindrical and cuboidal sub-sample to determine the sub-sample bulk density (g cm^−3^). We averaged the densities of sub-samples from the same bulk sample to calculate the sample's mean bulk density (g cm^−3^). We measured the compressive strength of one intact core (38-mm diameter, 50-mm height) from each bulk sample using an FX Test Pilot 250/300 Forney Press. We divided the pressure required to fail the concrete specimen by the top surface area of the core to calculate compressive strength (megapascals; MPa). This procedure closely matched standard concrete strength testing procedures (e.g., ASTM C39) except we used relatively small cores that we drilled out of pre-existing structures and cut to match our other sub-samples instead of using large, molded, test cylinders with greater height-to-diameter ratios. We obtained strength measurements below the normal range for concrete materials (14–43 MPa; [26]), but this is attributable to our modified testing procedure and does not indicate that our concrete samples were all below normal strength. In any case, our goal was not to determine the load-bearing capacities of the samples for quality assurance. Our goal was to obtain comparable measures of structural condition that we could statistically relate to other physicochemical and biological variables.

After pulverizing and sieving a sub-sample designated for biological analyses, we combined 10 g of its coarse fractions (>1 mm) with 20 mL of deionized water in a 50-mL centrifuge tube, vortexed the tube on medium speed for five minutes, incubated it at room temperature for 24 hours, then measured the pH of the solution with a calibrated digital pH meter after filtering it through coarse filter paper [27]. Unlike carbonation proportions, which depict the spatial extent of chemical change, these readings provided chemical descriptions of the concrete substrates as a whole.

### 2.4. Culture tests

To test the concrete endolith hypothesis, we performed a three-media culture test on each concrete sample. These tests, also known as growth media sterility tests, do not detect dead, inviable, or unculturable microbes. In a laminar flow hood, we poured several coarse fragments (1–5 mm) from each pulverized concrete sample onto three, 100 × 15 mm Petri dishes containing 25 mL of solidified malt extract agar (MEA), potato dextrose agar (PDA), or quarter-strength tryptic soy agar (TSA). We used coarsely pulverized concrete to inoculate the plates because finely pulverized concrete coated the growth media and interfered with colony formation. We shook the plates so that the small fragments rolled across the surface of the solid media, then dumped out any fragments that did not adhere to the agar. We sealed all plates with laboratory film to prevent desiccation, left them agar-side down for 24 hours at 25 °C, then dark-incubated them agar-side up for an additional six days. After the seven-day incubation, we photographed each plate and noted the presence or absence of visible microbial on the surface of the media. We also noted the level of visible microbial growth as minimal growth (with colony or other microbial growth formation estimated visually to be covering less than 15% of the growth agar's surface), or abundant growth (with microbial growth covering at least 15% of agar's surface). We selected these media types because they have general purpose, non-selective nutrient profiles that accommodate the growth of a wide variety of microbes. We used the MEA and PDA plates to cultivate any yeasts and molds, and we used the TSA plates to cultivate bacteria, although we noted any type of microbial growth on any of the plates as a positive life detection result, as long as there were no signs of contamination.

We used several controls during the culture tests to assess and manage contamination. We made all media plates in-house and in multiple batches, so before using plates from a batch, we incubated a subset of the batch while the plates were still sealed. We never opened these ‘batch controls’ since their initial preparation, so any growth in these plates indicated that the batch was possibly contaminated during preparation and thus unusable for testing. During inoculations, we prepared ‘workspace control plates’ by opening, manipulating, and resealing extra sets of plates in the same manner as the plates inoculated with concrete, except we left these plates empty. Growth on these plates suggested contamination during the inoculation steps, and we discarded all associated plates. We also inoculated plates with fragments of concrete samples that were autoclaved at 121 °C for 30 minutes three times over five days. These ‘sterilized concrete’ plates served as negative controls that displayed the physicochemical effects of concrete on the growth media without the influence of viable microbes. We took growth on these plates to mean that these and their associated plates were likely contaminated and unusable in the final test results. During final inspections, we discarded and replaced plates that looked compromised (e.g., had cracks or loose seals), had obvious signs of contamination (e.g., growth or discoloration inside the sealing film), and had growths emanating only from the edges of the plates (in case these resulted from contamination during plate handling).

### 2.5. Extraction and quantification of DNA

To test the concrete endolith hypothesis using biosignature molecules, we extracted double-stranded DNA (dsDNA) from our concrete samples. As in other studies that attempted to extract genomic DNA from troublesome substrates (e.g., [21]), we attempted several extraction techniques with streamlined, high-throughput DNA extraction kits that are popular and advantageous in microbiome research. Most notably, we attempted to extract DNA from finely pulverized concrete using the DNeasy PowerSoil Kit (12888-100; Qiagen) and ZymoBIOMICS DNA Miniprep Kit (D4300; Zymo Research) using their recommended procedures for low-biomass soil samples. We also used the kits with several procedural modifications including slightly increasing sample input mass, using the optimal bead beading settings, pooling lysates from 3–5 replicate samples into single extracts, heating the elution buffer, increasing the elution incubation time, repeating final elutions, and using a DNA clean and concentrator kit. Still, we did not recover detectable amounts of DNA from the vast majority of our samples. We suspected that typical lysis reagents and procedures may be unable break down hardened concrete. So, we investigated the usefulness of extended digestion/lysis/pre-treatment by visually observing the effects of EDTA and proprietary lysis solutions on concrete, similar to the decalcification observations made by [28]. We photographed small fragments from the Submerged-1 concrete sample before prolonged submersion in a prospective digestion reagent: 0.5 M disodium EDTA dihydrate, ZymoBIOMICSTM Lysis Solution (ZymoBIOMICS DNA Miniprep Kit), or Solution C1 (DNeasy PowerSoil kit) as well as in RO water and 5% acetic acid (for reference). We pressed the fragments into the undersides of 20-mm butyl rubber septa and photographed them using a Macropod Pro 3D system (Macroscopic Solutions) coupled with image stacking software (Zerene Systems LLC). Then, we placed the fragments and septa in 10-mL glass beakers and inundated each fragment with 7 mL of the treatment solution. We covered the beakers in film to prevent evaporation and left them undisturbed for 24 hours. After treatment, we re-photographed the fragments and searched for signs of decomposition.

Based on the results of the digestion experiment, we followed (with minor modifications) a non-kit-based protocol that involves an extended pre-digestion/pre-treatment lysis step that uses both EDTA and acetic acid. This DNA extraction protocol was posted by Kiledal and Maresca [29], and it was developed from previously successful methods [21],[22],[30]. For controls, we performed extractions simultaneously with five portions of sterilized glass beads (negative controls) and four portions of live topsoil (positive controls). We triple-sterilized the glass beads by 15-minute soakings in bleach with agitation, then 95% ethanol, followed by a 30-minute autoclaving. We briefly placed the sterilized beads in the pulverizer to mimic the pulverization process (but the beads were not pulverized). We used topsoil samples from Lubbock to represent local substrates with detectable amounts of microbial matter. However, we sampled soils of various conditions so that we could see a range of biosignature concentrations. Soil-1 was an agricultural soil sample that had been kept in lab storage for >3 years. Soil-2 was freshly harvested from a barren, dry, and shaded patch of urban ground. Soil-3 was freshly harvested from a gravel-covered footpath adjacent to irrigated urban gardens. Soil-4 was freshly harvested from an irrigated turfgrass area. We used the fine soil particles for DNA analysis after sieving all soil samples in the same manner as the concrete samples. We tested the sterility of the glass beads and the fertility of the soil with culture tests (described previously) and the ATP assays (described in the next section). In a laminar flow hood and using sterilized equipment, we placed 10 g of each sample into sterilized, 50-mL centrifuge tubes.

To each tube containing either concrete, sterilized glass, or live soil, we added a pre-treatment/lysis solution containing 5 mL of 0.5 M EDTA, 150 µL of 20 mg/mL nuclease-free water Proteinase K solution, 138 µL of 20% sodium dodecyl sulfate (SDS), and 200 µL glacial acetic acid. We incubated these tubes at 55 °C for 24 hours on a shaker unit set for gentle agitation (85 rpm). After incubation, we added 5 mL of nuclease-free water to each tube to increase the volume of supernatant. After vortexing at maximum speed for 10 minutes and centrifuging at 5,000 RPM for three minutes, we transferred the supernatants (~15 mL) into sterile tubes for DNA solubilization and binding. To each supernatant volume, we added 5 µL of 1-mg/mL yeast RNA solution and 125 µL of silica suspension (a silicon dioxide powder and water solution prepared by isolating medium-sized particles after large particles settled out after one hour and fine particles were pipetted out after 12 hours). We also added 30 mL of Qiagen Buffer QG amended with 25 mM sodium chloride solution and Triton X-100 surfactant. After mixing the solutions and allowing them to bind for 24 hrs at room temperature with gentle agitation, we centrifuged the tubes for five minutes at 5,000 RPM to pellet the silica-bound DNA. We decanted the supernatants and washed the silica pellets in 10 mL 80% ethanol to precipitate the DNA. After centrifuging for 30 minutes at 5,000 RPM at 4 °C, we decanted the supernatants and resuspended the pellets in 1 mL of 80% ethanol. We centrifuged the suspensions at 13,000 RPM for three minutes and pipetted off the supernatants. Then, we air-dried the pellets in a flow hood and resuspended them in 40 µL 10 mM Tris (pre-warmed to 60 °C) for five minutes and with gentle rotation. We centrifuged those suspensions for three minutes at 13,000 RPM and transferred the DNA-containing supernatants to sterile microtubes. We repeated this elution two more times to increase DNA recovery.

We concentrated our 120-µL DNA extracts by vacuum centrifuging (Savant Speed Vac Concentrator) for 4 hours at ~40 °C to dehydrate the volumes below 50 µL. We rehydrated the extracts with nuclease-free water back up to 50 µL to equalize the volumes so that we could accurately compare the yields between each of our DNA samples. We quantified the DNA using a Qubit® 3.0 fluorometer (Thermo Fisher Scientific) in ‘High Sensitivity dsDNA’ mode and following the manufacturer's instructions. We recorded the DNA concentration (ng of dsDNA µL^−1^ extract), then multiplied that by the extract volume (50 µL) to find the total recovered DNA per sample (ng). We divided that value by the mass of the original concrete sample (10 g) to obtain the sample DNA concentration (ng DNA g^−1^ concrete; a proxy measure of microbial abundance).

### 2.6. DNA amplification tests

We tested our DNA extracts for bacterial, fungal, and archaeal DNA using PCR amplification tests performed by RTL Genomics (Lubbock, Texas, USA). We selected universal primers to maximize the chances of amplification (28F: GAGTTTGATCNTGGCTCAG-519R: GTNTTACNGCGGCKGCTG for bacteria, ITS1F: TGGTCATTTAGAGGAAGTAA-ITS2aR GCTGCGTTCTTCATCGATGC for fungi, and Arch517F: CYTAAAGSRNCCGTAGC-Arch909R: TTTCAGYCTTGCGRCCGTAC for archaea). Like our culture tests, we noted which DNA extracts from which concrete samples had any successful amplification of microbial DNA and which extracts showed successful amplification of bacterial, fungal, or archaeal DNA specifically. We calculated the overall frequency of successful amplifications and the frequency of successful amplifications of each barcode type for all samples and among samples of each concrete category.

### 2.7. ATP quantification

We quantified ATP content of each sample using a rapid commercial and medical hygiene monitoring system. In a laminar flow hood, we inserted an UltraSnap™ Surface ATP Test Device (Hygiena) into each sample container. We tilted and rotated each container for 30 seconds so that its contents (coarse concrete fragments, coarse soil aggregates, or glass beads) touched the pre-wetted swab end of each device. We also swabbed the inner side and bottom walls of the containers for 30 seconds. After sampling, we immediately re-inserted the swab into the test device where it was inundated with a liquid-stable reagent containing luciferin and luciferase, which bind to ATP and luminesce. Immediately after swab inundation, we inserted the test device into a luminometer (SystemSURE Plus ATP Monitoring System, Hygiena). We recorded ATP contents Relative Light Units (RLUs), which is directly proportional to the amount of ATP collected by the test device.

### 2.8. Survey data analysis

We evaluated the concrete endolith hypothesis using results from the culture tests, DNA quantifications, amplification tests, and ATP assays. We determined if the frequency of positive results of a given test was greater than zero for concrete samples (or sub-groups of samples). We also compared DNA concentrations and ATP contents between our negative control group (*n* = 5), concrete sample group (*n* = 25), and positive control group (*n* = 4) using Kruskal-Wallis tests (α = 0.05).

We evaluated the ‘endolith-concrete interaction hypothesis’ by searching for ecological patterns in the life detection data. The culture tests produced ordinal data (no, minimal, or abundant growth on the culture plates) and the DNA amplification tests produced categorical results for each sample (amplification failed vs. amplification successful) and for each of the three test primers. To determine if endolithic communities in concrete are different among the general types of concrete found in urban landscapes, we noted if the frequencies of any growth on culture plates, abundant growth on culture plates, and successful amplification of microbial barcodes were unequal among the concrete categories. We also determined if these test results showed any patterns when the samples were sorted by continuous and non-repeating physicochemical parameters of the concrete (density, strength, pH) and then displayed as ordinal heatmap tables or categorical table matrices. We tested this hypothesis statistically by comparing DNA concentrations and ATP contents among the five types of concrete samples using Kruskal-Wallis tests (α = 0.05). To assess the relationships between concrete endolith community variables and concrete physicochemistry, we linearly regressed microbial DNA concentrations and ATP contents against concrete density, strength, carbonation proportion, and pH (α = 0.05). We performed all statistical tests using IBM SPSS Version 28.0.0.0.

### 2.9. Supplemental SEM

To explore microscopy-based detection of concrete endoliths, we examined one small, randomly selected fragment from the freshly pulverized portions of three concrete samples (Submerged-1, Belowground-3, and Ground-2) using a Hitachi S-4300SE/N SEM (Texas Tech University, College of Arts & Sciences Microscopy). In a laminar flow hood and using sterilized tools and equipment, we affixed each fragment to a 26-mm diameter, aluminum specimen holder using a double-sided carbon tape, and transported the mounted specimen to the microscope in a sealed, sterile container. To understand if concrete endoliths could be imaged in-situ and near to their natural state, we imaged the fragments uncoated, unstained, and without drying using the low-vacuum environmental scanning electron microscopy (ESEM) mode. As we traced the electron beam in a loose grid pattern across the surface of each fragment, we visually scanned the areas between 12–1200 µm^2^ for structures resembling intact cells or other forms of biological debris and saved the notable micrographs.

### 2.10. Supplemental identification of suspected fungal cultures

To confirm fungal taxa within our concrete samples, we inoculated MEA plates with fragments from three concrete samples (Submerged-1, Belowground-1, and Ground-2) before a 7-day incubation. We excised tissue from one fungal-like colony from each plate onto fresh plates, then, from the resulting pure cultures, excised and froze small pieces of tissue. We extracted genomic DNA from the tissues using the DNeasy Plant Mini Kit (Qiagen) following the protocol of Kaur et al. [31]. We prepared 25 µL-PCR reactions to amplify an internal transcribed spacer (ITS) gene region using the GoTaq Flexi Polymerase kit (Promega) and the universal ITS1OF/ITS40F primers (5′-TCCGTAGGTGAACCTGCGG-3′ as the forward primer and 5′-TCCTCCGCTTATTGATATGC-3′ as the reverse; [32]). We used the following thermocycler settings: initial denaturation step at 95°C for 4 min, 35 cycles of denaturation at 94 °C for 45 seconds each, annealing at 55 °C for 45 seconds, extension at 72 °C for 2 minutes, and extension at 72 °C for 10 minutes. We cleaned samples with amplicon types between 500–800 base pairs with the DNA Clean and Concentrator-5 kit (Zymo Research) and sent the reacted samples to the Yale DNA Analysis Facility (New Haven, Connecticut, USA) for Sanger sequencing. We trimmed the sequences at both ends by removing 25 bp sections that had more than 3 bases with phred scores <20 and ignored sequences shorter than 350 bp. We recorded the closest genetic match found in the Basic Local Alignment Search Tool (BLAST version 2.13.0; NCBI) and reported the taxonomic nomenclature displayed in the Taxonomy Database (NCBI).

## 3. Results

### 3.1. Physicochemical properties of concrete samples

The physicochemical properties of all 25 concrete samples are summarized in Table 2. Overall, the concrete samples showed considerable variation in physicochemistry, but the concrete categories that we considered did not always have distinct physicochemical properties. The bulk densities of our samples ranged from 1.3 to 2.4 g cm^−3^, but most of the poured chunks, slabs, and structures had similar densities around 2.3 g cm^−3^. As a category, CMUs had distinctly lower densities (x = 1.6 g cm^−3^) and were noticeably more porous than any other type of concrete. We tested compressive strength using a non-standard method, so the absolute strength readings were generally low, between 2–12 MPa (concrete generally tests above 14 MPa as it is being prepared for use). However, relative to each other, the readings were generally as expected in that the samples from the soundest structures (footings, bridges, and multi-level commercial buildings) had the highest compressive strengths (8–12 MPa) while the samples that were not designed for high standalone strength on a per gram basis (namely the CMU samples) had the lowest compressive strengths (2–4 MPa). In terms of carbonation, our samples ranged from having extremely shallow carbonation depths (> 1 mm; indicating that nearly all of the sample's internal volume had remained highly alkaline) to complete carbonation (in which the entire volume of the sample, including its center, had undergone a significant drop in alkalinity). Our more standardized expressions of carbonation depths, carbonation proportions, ranged from 0 to 1. Most sub-samples from the submerged, belowground, ground-level, and aboveground poured samples were <30% carbonated. In contrast, pre-cast CMU samples had a mean carbonation proportion of 0.73; most CMUs showed no highly alkaline regions in cross-section. Most concrete samples were highly alkaline (median pH = 11.8) when the samples were prepared as aqueous solutions. Similar to carbonation measurements, there was considerable variation in pH within concrete categories and no clear pattern except that samples of poured concrete had a noticeably higher mean pH (x = 11.7) than that of pre-cast CMU samples (x = 9.6).

**Table 2.** Physicochemical properties of 25 concrete samples from Lubbock, Texas, USA.

Bulk sample ID	Bulk density (g cm^−3^)	Compressive strength (MPa)	Carbonation proportion	pH
Submerged-1	2.32	8.239	0.292	9.90
Submerged-2	2.32	8.839	1.000	9.39
Submerged-3	2.44	3.447	0.091	12.28
Submerged-4	2.19	4.380	0.375	10.90
Submerged-5	2.36	7.272	0.000	12.45
Belowground-1	2.32	11.735	0.042	12.40
Belowground-2	2.29	7.641	0.000	12.10
Belowground-3	2.29	5.242	0.729	10.72
Belowground-4	2.33	4.443	0.165	12.61
Belowground-5	2.30	6.639	0.115	12.03
Ground-1	2.24	6.376	0.181	11.54
Ground-2	2.33	6.007	0.563	11.24
Ground-3	2.32	3.845	0.073	12.12
Ground-4	2.30	7.402	0.000	12.55
Ground-5	2.35	7.739	0.110	11.81
Aboveground-1	2.13	2.740	0.109	12.47
Aboveground-2	2.22	3.019	1.000	9.15
Aboveground-3	2.37	9.622	0.032	11.99
Aboveground-4	2.28	4.212	0.000	12.69
Aboveground-5	2.24	3.875	0.017	12.63
CMU-1	1.59	3.586	1.000	9.49
CMU-2	1.54	2.361	1.000	9.28
CMU-3	1.61	2.959	1.000	9.54
CMU-4	1.32	2.600	0.300	10.47
CMU-5	1.88	3.955	0.340	10.50

### 3.2. Culture test results

The frequencies of positive culture test results are summarized in Table 3. In alignment with the concrete endolith hypothesis, inoculum from 100% of our endo-concrete samples produced some visible microbial growth on at least one type of growth medium, and 68% of samples produced abundant growth on at least one medium. Growth appeared as small, isolated colonies and as widespread growths, often emanating from leftover concrete fragments but not always. The frequency of growth varied between the different media types; TSA plates had the highest proportion of plates with visible growth (92%), followed by PDA plates (76%) then MEA plates (64%). Abundant growth was most common on TSA plates (56%) but was fairly common on the MEA (48%) and PDA plates (40%). As predicted by the endolith-concrete interaction hypothesis, we noticed differences among the general types of concrete samples. All plates used to assay the CMU samples showed signs of growth (100% frequency), while 87%, 73%, 67%, and 60% of the plates inoculated with submerged underwater, belowground, aboveground, and ground-level samples showed signs of growth, respectively. The frequency of abundant growth also varied by concrete category. The plates with the CMU samples produced abundant growth most frequently (93% of plates), followed by submerged underwater samples (47%), aboveground samples (40%), belowground samples (33%), and ground-level samples (27%), indicating that the viability and culturability profiles of concrete varies among concrete types.

**Table 3.** Frequencies of visible microbial growth in culture test plates inoculated with various types of concrete.

	Percentage of plates with any visible growth			Percentage of plates with abundant growth		
Concrete category	*MEA*	*PDA*	*TSA*	*MEA*	*PDA*	*TSA*
Submerged fragments (poured)	80*	80	100	60	40	40
Belowground structures (poured)	60	60	100	20	20	60
Ground-level slabs (poured)	40	80	60	20	20	40
Aboveground structures (poured)	40	60	100	40	40	40
Concrete Masonry Units (pre-cast)	100	100	100	100	80	100
All concrete samples	64	76	92	48	40	56

Note: Culture test plates contained malt extract agar (MEA), potato dextrose agar (PDA), or tryptic soy agar (TSA). Following incubation, we assessed the plates as having no growth or any growth, and we noted which plates had abundant growth (growth covering >15%). We report the percentage of concrete samples in a given concrete category that displayed such growth on each media type. *Example data point that can be interpreted as: 80% of MEA plates (4 out of 5 plates) inoculated with samples of submerged concrete showed microbial colony growth after the 7-day incubation period.

Moreover, culture test results showed some patterns when sorted by some physicochemical variables (Table 4). Abundant growth results were generally more common for the least alkaline and least dense concrete samples. Yet, culture results did not vary clearly by concrete compressive strength.

**Table 4.**
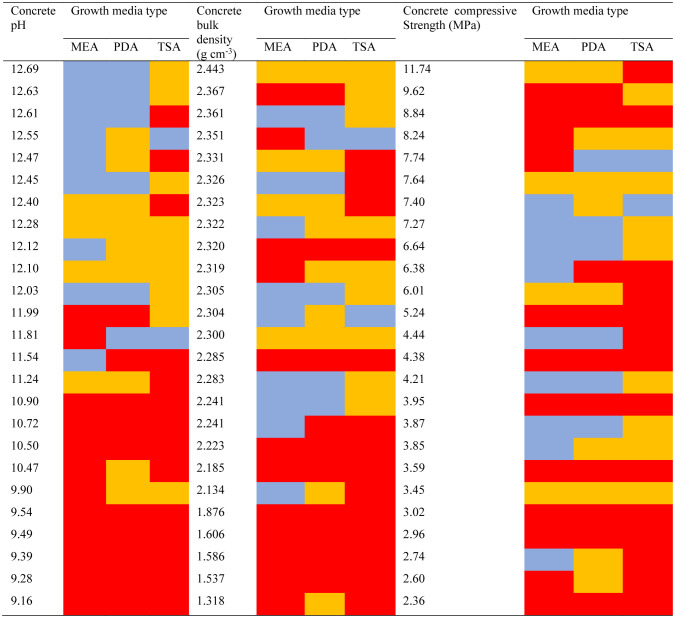
Culture test results sorted by the properties of the associated concrete samples.

### 3.3. DNA quantification results

We observed that 24-hour soaking in 0.5M EDTA, ZymoBIOMICS™ Lysis Solution, and 5% acetic acid induced micro-cracking and some mineral dissolution in small concrete fragments (Figure S1). Submersion in reverse osmosis water and Solution C1 (from the DNeasy PowerSoil kit) did not induce structural changes in the treated concrete fragments (photographs not shown).

Observing that EDTA and acetic acid can partially digest concrete samples, we followed a direct DNA extraction protocol that involves pre-treating the concrete in a solution containing both EDTA and acetic acid (as well as several other reagents) for an extended period of time (i.e., [29]). We isolated quantifiable amounts of dsDNA from all 25 concrete samples. Moreover, DNA concentrations were statistically different among the sterilized negative control samples (x = 0.1 ng µL^−1^), concrete samples (x = 5.7 ng µL^−1^), and live soil positive control samples (x = 45 ng µL^−1^; *p* = 0.003). The ranking of mean DNA concentrations among these general treatment groups matched our prediction that endo-concrete substrates host low-biomass microbial communities, but other observations confounded these results. The post-hoc pairwise comparison of concrete samples and negative controls revealed only a marginally significant difference (*p* = 0.051), and some concrete extracts measured lower than some extracts from sterilized control samples, indicating that some concrete samples had less DNA than what would be expected from background contamination. Still, several concrete samples yielded 2-172 times more DNA than any negative control sample.

**Figure 2.**
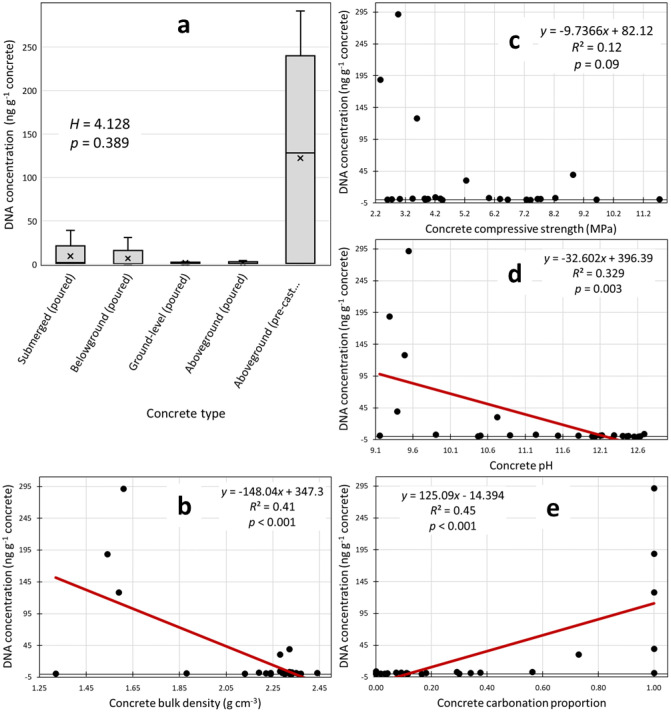
Variations in microbial DNA concentration among concrete samples. Note: We analyzed DNA concentrations in concrete samples of different categories (a), and among concrete samples of various densities (b), strengths (c), alkalinities (d), and carbonation proportions (e). A non-significant but noteworthy comparison of DNA concentrations is shown for the five types of concrete (a). Significant linear regressions are shown for DNA concentrations versus continuous physicochemical variables (b, d, e).

We found significant relationships between microbial DNA concentrations and some physicochemical properties of the host concrete and noteworthy variations in DNA among the five types of sampled concrete (Figure 2). DNA concentrations in concrete decreased as concrete density increased (Figure 2a), increased as extent of the carbonation increased (Figure 2b), and decreased as the overall pH of substrate increased (Figure 2c). However, the *R*^2^ values show that any one of these physicochemical variables only explained about 30–45% of the variation in endogenous DNA concentrations. The relationship between DNA and compressive strength was not significant (*p* = 0.09). We compared DNA concentrations among the five types of sampled concrete and found no significant differences (*p* = 0.389). However, we noticed that the highest DNA yields (between 100–300 ng dsDNA g^−1^ concrete) were all from CMU samples while no samples from any other concrete type exceeded 40 ng dsDNA g^−1^ concrete (Figure 2d).

### 3.4. DNA amplification test results

As predicted by the concrete endolith hypothesis, over half of our DNA extracts contained DNA that was PCR-amplifiable using universal microbial primer pairs (Table 5). All of the concrete DNA extracts that contained amplifiable DNA had dsDNA concentrations higher than 0.2 ng µL^−1^ while all extracts with lower concentrations failed to amplify. Bacterial DNA was most commonly amplified (from 14 of 25 samples), followed by archaeal DNA (5 samples), then fungal DNA (4 samples). For many samples, bacterial DNA was the only type of DNA to amplify, but all of the samples that contained archaeal and/or fungal DNA also contained bacterial DNA. All of the negative control extracts failed to amplify while all of the positive control extracts amplified all three types of target DNA. As predicted by the endolith-concrete interaction hypothesis, we noted differences in amplification patterns among the various categories of concrete (Table 5). Amplification of any kind was most common for the CMU samples and submerged samples and rarer for the belowground, ground-level, and aboveground samples. In terms of particular primer pairs, archaeal and fungal DNA was most commonly detected in CMUs, followed by submerged and belowground samples; no DNA was amplified from ground-level and aboveground samples using the archaeal or fungal primer pairs. We most commonly detected bacterial DNA in CMU and submerged samples, but some samples from all other concrete categories contained bacterial DNA.

**Table 5.** Percentage of concrete samples from which DNA was extracted and amplified.

	Type of DNA targeted for amplification		
Concrete Category	Archaeal	Fungal	Bacterial	Any DNA amplification
Submerged fragments (poured)	20*	20	80	80
Belowground structures (poured)	20	20	40	40
Ground-level slabs (poured)	0	0	40	40
Aboveground structures (poured)	0	0	40	40
Concrete Masonry Units (pre-cast)	60	40	80	80
All concrete samples	20	16	56	56

Note: We attempted DNA amplifications with the following primer pairs: Arch517F/Arch909R (archaeal), ITS1F/ITS2aR (fungal), and 28F/519F (bacterial). We report the percentage of concrete samples in a given concrete category that yielded DNA that could be amplified with each type of PCR primer as well as all primers collectively. *Select data point that can be interpreted as: 20% of DNA extracts (1 out of 5 extracts) obtained from submerged concrete samples contained DNA that was amplified using the archaeal PCR primer.

Amplification results seemed to vary only loosely with concrete physicochemistry (Table 6). Amplifiable bacterial DNA was obtained from concrete samples that ranged widely in terms of pH, density, and compressive strength. Interestingly, bacterial DNA was detected among the most alkaline, dense, and structurally strong samples. We did not detect archaeal and fungal DNA in concrete samples with pH readings greater than 10.9, but we did detect such DNA among the samples with high densities (>2.3 g cm^−3^) and high compressive strengths (>8.8 MPa).

### 3.5. ATP assay results

We detected ATP biosignatures in 48% of concrete samples, a positive overall life detection result. On average, we detected 3.6 RLUs of ATP in concrete samples, which is between the sterilized glass negative control samples (all had 0 RLU) and the live soil positive control samples (x = 288 RLUs). However, as with DNA concentrations, there was considerable variation within the concrete sample group. We did not detect ATP for 13 of the 25 concrete samples, while seven samples had ATP readings between 1–2 RLUs, and three samples had ATP readings between 7–42 RLUs. That is, most concrete samples were as sterile as the sterilized controls, but several had more than would be expected from sterile substances, and the two most ATP-rich concrete samples (27, 42 RLUs) overlapped with the two least fertile soil samples (Soil-1 with 21 RLUs, Soil-2 with 34 RLUs). However, we did not statistically test for significant differences between negative control, concrete, and positive control samples because there was no variation in the negative control group, and the values in the concrete group were steeply skewed.

**Table 6.**
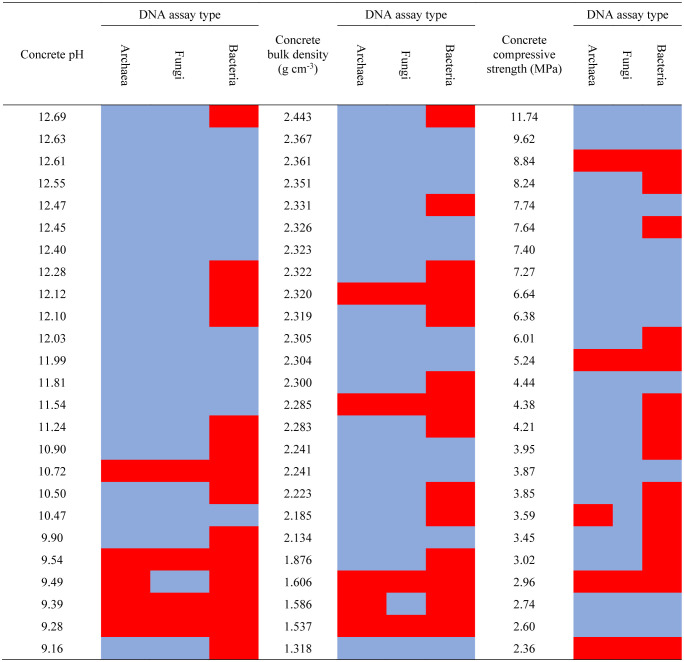
DNA amplification results sorted by the properties of the associated concrete samples.

**Figure 3.**
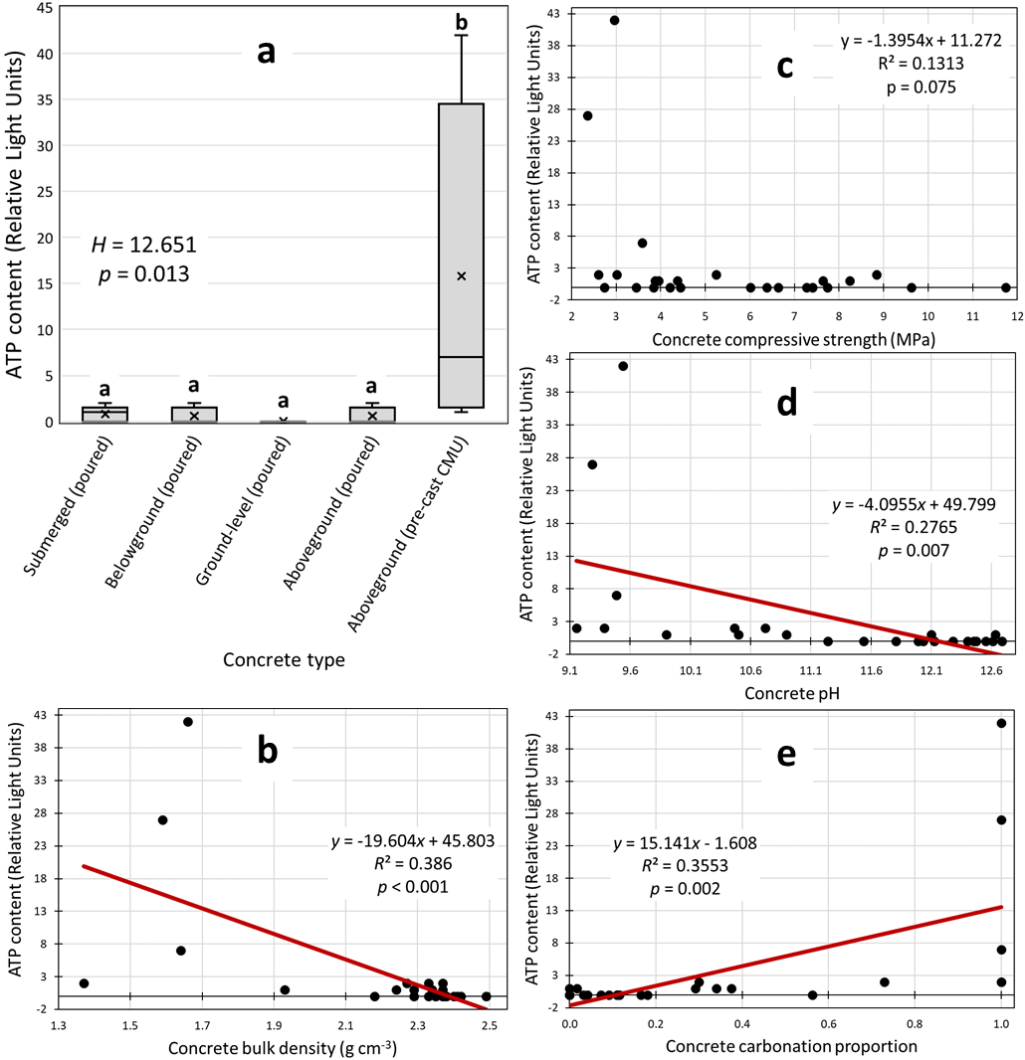
Variations in microbial ATP content among concrete samples. Note: We analyzed ATP contents in concrete samples of different categories (a), and among concrete samples of various densities (b), strengths (c), alkalinities (d), and carbonation proportions (e). A significant comparison of DNA concentrations is shown for the five types of concrete (a); different letters indicate significant differences among groups. Significant linear regressions are shown for ATP contents versus continuous physicochemical variables (b, e).

We found significant relationships between ATP readings and the same concrete physicochemical properties that predicted DNA concentrations, as well as significant differences in ATP among the five types of sampled concrete (Figure 3). We compared ATP concentrations among the five types of sampled concrete and found that submerged, belowground, ground-level, and aboveground poured structures had similarly low ATP concentrations (0–2 RLUs), while ATP in the aboveground CMU group was significantly higher than all other groups (Figure 3a). ATP content in concrete generally decreased as concrete density and strength increased (Figure 3b and c), increased as extent of the carbonation increased (Figure 3b), and decreased as the overall pH of substrate increased (Figure 3c). We inferred from the *R*^2^ values that each of these physicochemical variables explained about 30% of the variation in ATP content, although not all relationships were significant.

### 3.6. Electron microscopy results

**Figure 4.**
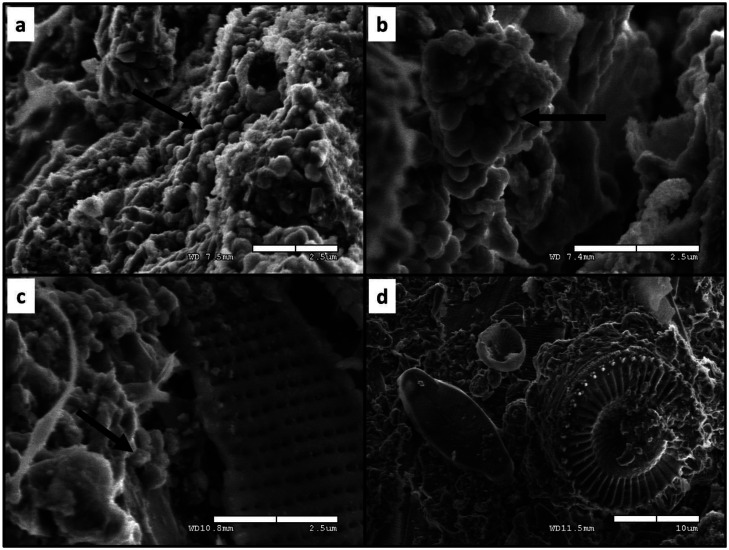
SEM micrographs of cell-like structures and biological debris embedded inside concrete. Note: (a) belowground concrete from a buried post setting, (b) concrete from a ground-level housing pad, and (c, d) concrete that was permanently submerged in a freshwater stream. Panels a and c show the portions of the three concrete samples that include suspected endolithic microbes (the black arrows point to exemplary cells-like structures). Panels c-d also show the remains of diatoms. Working distances and scale bars are displayed in the lower-right of each panel.

We obtained ESEM micrographs of what appear to be intact microbial cells embedded in the concrete substrates (Figure 4). Although each fragment appeared solid and relatively smooth to the unaided eye, we observed a great deal of microscopic topography and surface variation across all fragments. Much of the visible surface of any given sample seemed devoid of anything biological. However, during inspections, we came across isolated sections that appeared to be covered with cell-like structures, often clumped near other cell-like structures (Figure 4a–c). The fragment from the submerged concrete sample had recognizable diatom protists visible in the vicinity of the cell-like structures (Figure 4c–d). These silica-based frustules of pennate and centric diatoms were embedded and scattered in the concrete along with the more prokaryote-looking cell-like structures.

### 3.7. Taxonomic assignment of fungal cultures

Although we visually recognized both bacteria and fungi among the cultures grown during the culture tests, we confirmed that fungi are among the viable microbes existing within ordinary concrete. From three concrete samples, we isolated distinct fungal culture whose ITS DNA was matched closely to sequenced fungal species (Table 7). According to the matching organisms, the fungi we isolated are only distantly related, and each isolate is a member of a different fungal class.

**Table 7.** Fungal species cultured and isolated from within three concrete samples.

Sample ID	Description of closest genetic match						
	Accession	Phylum	Class	Order	Family	Genus	Species
Submerged-1	KX067847	Ascomycota	Sordariomycetes	Hypocreales	Nectriaceae	*Fusarium*	*(unassigned)*
Belowgrnd-1	KX099664	Ascomycota	Dothideomycetes	Pleosporales	Pleosporaceae	*Alternaria*	*alternata*
Ground-2	KM249084	Mucoromycota	Mucoromycetes	Mucorales	Rhizopodaceae	*Rhizopus*	*arrhizus*

Note: We extracted DNA from the pure fungal cultures, amplified the internal transcribed spacer DNA (ITS1, ITS4), analyzed the amplified DNA with Sanger sequencing, and found the closest sequence match using the Basic Local Alignment Search Tool (BLAST; NCBI). All matches had E values of 0.0 and percent identities of >99.6.

## 4. Discussion

### 4.1. Endoliths may be common constituents of concrete

We believe our results are sufficient to claim that concrete is frequently inhabited by live microbes. All of our endo-concrete samples contained at least some viable microbes (Table 3), suggesting that microbes can remain viable inside most ordinary concrete. Yet, we are uncertain if the microbes that reproduced during the culture tests were active or dormant while inside concrete. Endoliths in natural rocks are often in cryptobiotic states (e.g., spores), which helps facilitate long-term survival in harsh, lithic environments [33],[34]. These tests also indicated that many endo-concrete microbes are easily cultivable, a trait that opens up several other possibilities for study and manipulation. For example, we followed our culture tests with some colony isolations and genetic analyses to provide, to our knowledge, the first evidence that fungi are among the incidental endolithic microbes in ordinary concrete (Table 7). And, though it was not our main objective to conduct detailed taxonomic or phylogenetic analyses, those genetic analyses suggest that we can expect endo-concrete fungi from several classes and phyla, just as Maresca et al. [21] found high-level diversity among endo-concrete bacteria. Overall, our culture tests demonstrated high sensitivity but mainly provided simple qualitative or categorical presence/absence data of culturable microbes, and it was our underlying goal to test the concrete endolith hypothesis from several perspectives (*sensu* [35],[36]).

We also used molecular biosignatures to test our first hypothesis. Biomolecules like nucleic acids, proteins, metabolites, and pigments are used often as quantifiable biosignatures in life detection missions [37],[38]. While DNA data does not indicate the viability or culturability of the microbes, we used DNA as a biosignature primarily because DNA, when successfully extracted and quantified, infers microbial abundance (e.g., biomass; [39],[40]. Here, we used the extraction protocol of Kiledal and Maresca [29] with fair success and calculated dsDNA concentrations per gram of concrete for each sample. We extracted and detected significant amounts of dsDNA from several concrete samples, thus supporting our first hypothesis.

DNA extraction also allowed for more specific tests that individually screened for bacteria, archaea, and fungi within our samples while simultaneously determining if the extracted DNA could be amplified via PCR and made usable for subsequent analyses. After all, isolating enough DNA to be detectable does not necessarily mean the DNA is of high quality and amenable to PCR-amplification [41], partly because DNA can be degraded in several ways that inhibit amplification [42]. If DNA amplification cannot be achieved, the overall value of direct DNA extractions would be severely reduced, not necessarily for preliminary life detection studies such as this, but certainly for any work investigating more specific microbiome parameters (e.g., species diversity and composition). Our amplification attempts revealed archaeal, fungal, and bacterial DNA from several concrete samples (Table 5). This further supports our claim that some incidental concrete endoliths are fungi and archaea. Archaea are commonly detected among endoliths in natural rock formations [43]. Bacterial DNA was most commonly amplified among our concrete samples and, for several samples, was the only type of DNA to amplify. Also, all the samples with amplifiable fungal or archaeal DNA were never without amplifiable bacterial DNA, suggesting that bacteria are collectively adapted to a wider range of endo-concrete environments and/or bacteria must be present for other microbes to become abundant; further trans-domain ecological investigations are needed (e.g., [44]).

We used ATP as another molecular biosignature with moderate success. Bioluminescence-based ATP quantification systems, such as the one used here, are popular because they offer rapid detection of a wide range of biological contaminants [45]. And though these systems may not be ideal for industry-wide quality control purposes and high-stakes sterility testing [46], we reasoned that these could indicate the relative amounts of microbial matter among samples in one experiment. Our ATP results were similar to DNA quantity results in that many concrete samples overlapped with the negative controls (with readings of 0 RLUs) while several others had low but detectable amounts of ATP, and a couple had ATP contents comparable to some soil samples. This also supports our first hypothesis, and these ATP data might have the additional advantage of indicating not only microbial abundance but also microbial activity [47], presumably because ATP is fundamental to cell energetics, but it is unclear from our data if microbial metabolisms, reproductive rates, etc. varied among samples. The main contribution of ATP data here was corroborating our DNA data; ATP and DNA quantities were strongly positively correlated (*R* = 0.97).

Finally, we found SEM to be a useful tool for detecting concrete endoliths. Microscopy was not part of our primary survey, but we acknowledged that the concrete endolith hypothesis could benefit from micrographs, which are incredibly valuable as visual microbial biosignatures, especially when used in conjunction with other life detection tools [48]. There are ways to enhance the capabilities of SEM, but we thought it worthwhile to attempt visualization without significant sample manipulation or advanced instrument settings (i.e., with only low-vacuum ESEM and without stains or coatings). We discovered small areas with prokaryote-like structures in all three samples (Figure 4), perhaps arranged in biofilms, which supports our first hypothesis. In one sample of submerged concrete, we saw diatoms in the vicinity of the cell-like structures (Figure 4c–d). We doubt the diatoms were actively living in the concrete and presumed that we discovered remnant frustules, perhaps frustules that were present among the aggregates used to manufacture this concrete or were flushed into the endo-concrete environment by water moving microscopic cracks in the concrete. Still, this displays non-living biological/organic debris within concrete, which may facilitate the development of endolithic ecosystems [49],[50].

### 4.2. Concrete endolithic communities are highly variable

Our endolith-concrete interaction hypothesis considered that modern cities hold an unaccountable variety of concrete structures. Depending on its unique features, a concrete structure may support rich microbial communities *or* be effectively devoid of life. Our stratified sampling of concrete types ensured that our final set of samples captured some of the incredible diversity of concrete within our study area. As predicted, we observed a wide range of endolithic life detection results among those concrete structures.

The five concrete categories showed noteworthy dissimilarities in the culture tests (Table 3), DNA quantifications (Figure 2d), amplification tests (Table 5), and ATP quantifications (Figure 3d). The aboveground CMU sample group consistently yielded the most evidence of microbial life. While being positioned aboveground may not independently promote endolith establishment, being pre-cast into hollow-style forms with high surface areas may make CMUs more open to microbial colonizers and life-sustaining inputs of matter and energy. We suspect that CMUs in states of extreme physical weathering positioned in ecologically active areas (e.g., CMU-2, CMU-3) may even be able to support endolithic establishment by multicellular microbiota, but we did not specifically screen our samples for non-fungal eukaryotes. Submerged concrete also had a preponderance of positive life detection results compared to the remaining concrete categories. While the submerged samples were dense and mostly impermeable to water, long-term positioning underwater may buffer concrete from climatic extremes and/or inundate the concrete with waterborne microbes and organic matter that can be eventually incorporated in the interior portions of the concrete. The diatom remains we micrographed in a submerged concrete sample may represent a type of organic matter input received only by submerged concrete structures but may also exemplify the importance of environmental setting. Belowground, ground-level, and aboveground samples also yielded positive life detection results (e.g., Tables 4 and 6) but less consistently within those categories. Overall, the categories we developed were useful in demonstrating variability in endolithic communities inside concrete, but we also found relationships between life detection results and concrete physicochemical variables.

Lower concrete densities seem to favor microbes (Tables 4 and 6, Figures 2a and 3a). Bulk density is inversely related to porosity, so we presume our least dense samples retained more bioavailable water and contained more space for potential endoliths [51]. More direct measurements of porosity and permeability would help resolve this relationship as would inspections of extremely porous and dense concrete. Concrete density is positively related to strength [52], but strength measurements predicted much less of the variation in biological variables (Tables 4 and 6, Figures 2b and 3b). This may be because strength is not a purely physical property but a complex mechanical measurement that encompasses other factors [53], many of which may be irrelevant to microbial survival. Our failure to detect a clear relationship between the presence of microbes and concrete strength means that natural levels of endolithy may not be very pertinent to concrete engineering. However, these results do not discount the use of certain microbes as structural bioindicators [21] or the well-documented role of surface-dwelling microbes (epiliths) in the biodegradation of concrete [54],[55].

Biological variables often correlated with concrete pH. Our categories of culture growth (*none*, *minimal*, and *abundant*) are somewhat arbitrary, and the amplification result categories (*failed* vs. *successful* amplification) are purely qualitative. Yet, when sorted by sample pH, instances of abundant culture growth (Table 4) and successful DNA amplification (Table 6) seem to indicate a concrete pH threshold of around 11, below which endolithic communities may become much more diverse and contain more viable cells. The quantitative regressions of pH versus DNA yields (Figure 2c) and ATP contents (Figure 3c) showed similar results. These findings are intuitive given that substrate pH is a fundamental factor controlling environmental microbes [56], and extreme pH readings above 11 may exclude many potential endoliths.

Another pH-related variable, carbonation proportion, similarly predicted DNA yields (Figure 2b) and ATP contents (Figure 3b). Carbonation is complex, but it is generally considered a degradative process that gradually reduces concrete durability by increasing its permeability and porosity [57]. Our results suggest that carbonated concrete is more favorable to microbes, perhaps because carbonated concrete is not extremely alkaline and/or endolith establishment is promoted by the same factors that promote carbonation (e.g., time, cracking, higher surface area; [58],[59]. Further study is needed to determine if microbes are more abundant in carbonated zones and if feedbacks occur between carbonation reactions and endolithic microbes.

Our main survey showed inter-sample variation, supplemental SEM inspections of concrete samples revealed some intra-sample variation. As we scanned fragments from three concrete samples, we noticed that evidence of live microbes was highly localized within the host concrete and much of the visible area appeared barren. This is reminiscent of microbial ‘hotspots’ sometimes observed in soils [60]. Broader and more systematic use of microscopy (e.g., [20]) might reveal that internal substrate architecture controls endolith distribution and ultimately community function [61].

### 4.3. Accounting for false negatives and false positives

Life detection studies can be confounded by several factors. Neveu et al. [62], discussing life detection in astrobiology, described the various intricacies of confirming microbial life without the privilege of more direct evidence. When searching for life inside terrestrial concrete, the burden of proof is not as heavy as it would be when investigating extraterrestrial life. However, verifying instances of endolithy is rarely straightforward. The low levels of biomass and biological activity, combined with the fact that the organisms are entirely inside a solidified substrate, make it difficult to discern living microbes and rule out false positives produced by past lifeforms [63], purely abiotic features [64], or contaminants [30],[65]. As with other studies, our chief concern was contamination leading to false positives–a risk that is perhaps impossible to eliminate completely but nonetheless deserves careful consideration [35],[42]. False negatives produced by insufficient information or inadequate methods are also concerning [66]. With all this in mind, we carefully considered all methodological stages, from field sampling to benchtop procedures, so as to maximize detection rates and minimize contamination risks.

We examined concrete samples found in the field (not experimentally prepared), and all had unique sizes and shapes, so we relied on a drill press corer and tile saws to extract usable sub-samples out of our bulk samples. These tools are powerful enough to manipulate solid concrete but are not intended to carry out microbiologically sensitive tasks. Attempting to clean these power tools was difficult and time consuming, but these tools allowed us to (1) definitively isolate the internal portions of each sub-sample and cut off any surface portions, (2) control the final dimensions of the sub-samples, and (3) know precisely from what part of the bulk samples we obtained the sub-samples. Moreover, it was easy to apply sterilizing ethanol and flaming to the large, solid sub-samples (a surface sterilization technique that would be inappropriate for small or granular samples with larger surface area-to-volume ratios). Before analysis, most of our sub-samples required pulverization, which exposes the internal portions of a solid sample, making the sample vulnerable to environmental contamination as well as direct contamination from the pulverizing instrument [67],[68]. Our pulverization method was a compromise between being able to process large samples to certain particle sizes (using a custom-made pulverizer) and managing contamination (by using various methods to decontaminate the samples, the instruments, and the immediate workspace).

Contamination was not a major concern during our physicochemical measurements, but we carefully considered false readings during life detection procedures. Culture tests are highly sensitive life-detection tools, and in any given test, a huge diversity of microbes can be reliably detected using a small set of non-selective media types [69]. Unspecific media is preferred when searching broadly for microbial life, but this increases the risk of cultivating contaminant microbes [70]. Considering this, we designed our culture tests to be straightforward with a very direct inoculation method, somewhat similar to Onofri et al. [71], but we used several types of controls and criteria to filter out suspicious growth plates that could lead to false positives. Unscheduled inspections of our negative control plates (inoculated with sterilized concrete) also revealed that some concrete substrates seem to react chemically with some growth media, producing rapid changes in the media that could be misconstrued as biological growth. We accounted for this type of false positive, but this could be investigated further because reactions between samples and growth media might affect microbial growth [72].

We encountered some problems with contamination during DNA testing; our negative control samples yielded very low but detectable amounts of DNA. We assume that DNA was from contaminant microbes, possibly from the silicon dioxide used during the extractions. That powder had high chemical purity, but it was the only material that we did not decontaminate ourselves nor could we presume it to be pre-sterilized or self-sterilizing. Fourteen of 25 concrete samples overlapped with the negative controls in terms of DNA yield. This could mean that many of our concrete samples had undetectably low amounts of endogenous DNA and their final DNA readings reflect background contaminants. Alternatively, and despite some precautions, the carbonate- and calcium-rich concrete may have inhibited the DNA extraction, artificially reducing DNA yields to negligible levels and producing false negatives [73]. In any case, it is now more doubtful that any problems with extraction lie in the pre-digestion/lysis steps. Our auxiliary digestion experiment indicated that concrete, despite its unique physicochemistry and recalcitrance, is visibly degraded by typical lysis reagents and solutions (Supplemental Figure 1). So, when combined with pulverization and vortexing, a pre-digestion step should help ensure that endolithic microbial matter is at least liberated from the concrete. Furthermore, we conclude that, even with effective lysis solutions, commercial DNA extraction kits designed around small sample inputs (0.25–1 g) are not suitable for endo-concrete samples because there is so little microbial biomass inside most concrete. Methods that accommodate large sample inputs, such as the 10 g sample inputs instructed by Kiledal et al. [29], may be the only way to overcome the low-biomass issue. In the end, problems with the other stages of DNA extraction and quantification may make DNA a less sensitive life detection tool than culture tests. Still, DNA isolations open up several other analytical possibilities, assuming the DNA can be amplified.

We could not amplify DNA from our negative control extracts while the positive control extracts showed amplification by all test primers. Thus, we did not encounter any obvious signs of contamination, but PCR can amplify even trace amounts of contaminant DNA along with endogenous DNA, a typical problem for low-biomass environmental samples [74]. Though outside the scope of this study, later sequencing of the amplified DNA may allow for statistical decontamination procedures, as demonstrated by Kiledal et al. [22], but it is unclear if such approaches will be suitable for studies examining large and diverse sets of found concrete samples. Concrete may also be a difficult environmental sample because all microbes inside ordinary concrete are, in a sense, contaminants because no ordinary concrete is designed to harbor microbes. Any microbes found within concrete originated from any number of environmental microbes that were incidentally entrapped in the concrete during its formation [22] or secondarily colonized the concrete after it had solidified [20]. In any case, we will be better able to disentangle DNA-based contamination as more metabarcoding and metagenomic data for concrete endoliths accumulates.

Unlike DNA extraction and quantification, our ATP procedure was simple and rapid, so the risk of contamination was relatively low. In fact, we suspect that we obtained more false negatives in these results (i.e., readings of zero ATP when ATP is present). We believe that our assay method could be improved in several ways to be more standardized and sensitive. For example, instead of timed swabbings of dry concrete fragments, analyzing concrete solutions (similar to solutions prepared for pH analysis) may offer more standardization (if solutions are consistently prepared) and sensitivity (if swabs optimized for fluids and/or low-biomass samples are used).

## 5. Conclusions

This study gathered compelling evidence that the insides of ordinary concrete are often inhabited by various microbes, thereby revealing a novel microbial guild and a new type of urban microbiome. We corroborated the few studies that previously demonstrated microbial life within ordinary concrete [20]–[22] and made several new contributions to the understanding of concrete endoliths. Our results broadly support the emerging hypotheses that concrete is potential habitat for endolithic communities and those communities vary according to the environmental conditions imposed by the concrete. This variation, ranging from negligible microbial levels to microbial levels on par with low-biomass soils, is an important caveat and aligns with the well-developed idea that lithic substrates and building materials vary in terms of their ‘bioreceptivity’ [75]–[77]. This sort of heterogeneity also makes concrete analogous to urban soils, which can vary considerably within a given city because of various human land uses [78],[79]. Concrete alkalinity and permeability seem to be among the most important physicochemical factors affecting unintentional endolithy, but bulk forms and environmental settings also seem important.

There are many options for future work in this area, but the most important questions can be answered by refining life detection methods, exploring new methods, conducting more in-depth microbiome analyses, and analyzing other types of concrete samples. Methodological refinements (e.g., more efficient sample preparation methods) will minimize false results, and new methods (e.g., cell enumeration, assays of biomolecules other than nucleic acids and ATP, respiration assays) will elucidate other aspects of concrete endolith communities (e.g., biovolume, trophic structure, activity rates). Microscopy may be especially suited to differentiating the different types of endoliths recognized by Golubic et al. [80] and Marlow et al. [81]. Metabarcoding and -omics studies, especially those encompassing fungi and archaea, would reveal the true level of taxonomic and functional diversity of concrete endoliths.

Such studies could also pinpoint incidental microbes with physiologies that either degrade concrete (e.g., silica solubilization [82]) or strengthen concrete (e.g., calcite precipitation) and microbes that serve bioindicators as structural indicators [21],[22]. Also, there remains a need to investigate other concrete variables such as mix type, amendments used (admixtures, reinforcements, coatings, etc.), positioning (indoor vs. outdoor, in different climate zones, etc.), smaller-scale spatial variables (endolithic zones near the surfaces of concrete vs. deep endolithic zones) and temporal variables (age, life cycle stage, etc.). The correct experimental designs could also help pinpoint the origins of concrete endoliths, find links between concrete endoliths and epiliths, and reveal interactions between incidental endoliths and intentionally added, ‘autoendolithic’ microbes in self-healing concrete.

The ultimate goal is to reevaluate concrete's position in the biosphere. Some have articulated concrete's role in large-scale phenomena such as environmental modification, planetary urbanization, and the global proliferation of built environments [83],[84]. And Earth's cumulative supply of concrete has even been cited as a definitive marker of our transition into the Anthropocene [2],[14]. Concrete endoliths represent yet another dimension to consider when trying to understand concrete's effect on biodiversity, its carbon footprint, and its overall role in global change.

## Supplementary materials

**Figure S1.**
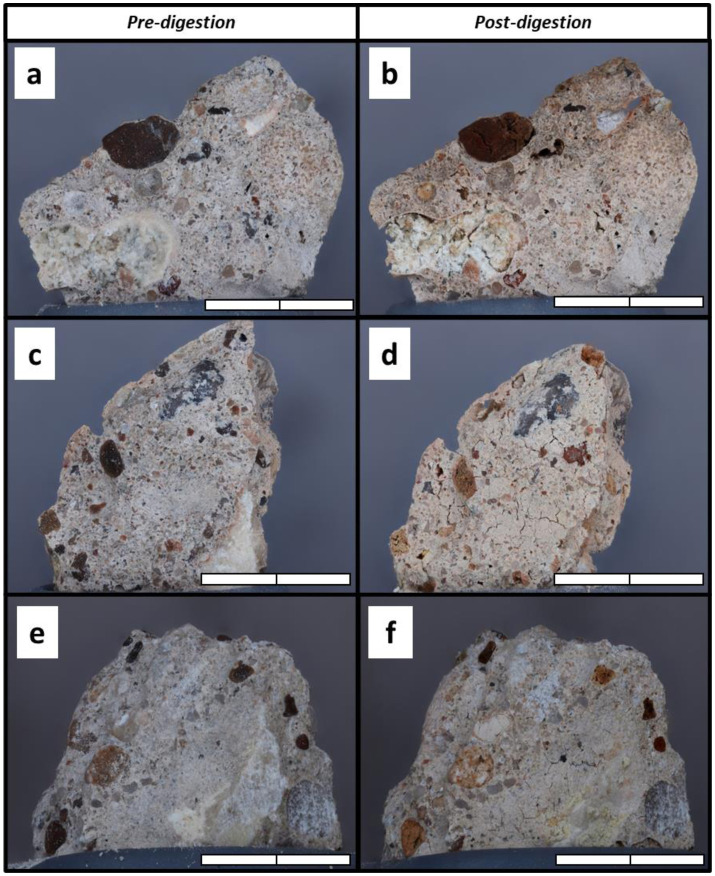
Concrete fragments before and after submersion in digestion reagents. Note: We assessed the utility of EDTA (panels a-b), acetic acid (c, d), and ZymoBIOMICS Lysis Solution (e, f) as digestive, pre-treatment liquids during the extraction of microbial DNA from concrete. We submerged fragments from the insides of a concrete sample in the treatment solutions for 24 hrs and compared the pre-treatment imagery of each sample (left-side panels) to its associated post-treatment imagery (right-side panels). Note the post-treatment cracking and mineral dissolution. 5 mm scale bars are shown in the bottom-right corner of each panel.
